# Testing of Alignment Parameters for Ancient Samples: Evaluating and Optimizing Mapping Parameters for Ancient Samples Using the TAPAS Tool

**DOI:** 10.3390/genes9030157

**Published:** 2018-03-13

**Authors:** Ulrike H. Taron, Moritz Lell, Axel Barlow, Johanna L. A. Paijmans

**Affiliations:** Institute for Biochemistry and Biology, University of Potsdam, Karl-Liebknecht-Str. 24-25, 14476 Potsdam, Germany; moritz.lell@alumni.uni-potsdam.de (M.L.); axel.barlow.ab@gmail.com (A.B.)

**Keywords:** ancient DNA, short-read mapping, palaeogenomics, paleogenomics, alignment sensitivity/specificity

## Abstract

High-throughput sequence data retrieved from ancient or other degraded samples has led to unprecedented insights into the evolutionary history of many species, but the analysis of such sequences also poses specific computational challenges. The most commonly used approach involves mapping sequence reads to a reference genome. However, this process becomes increasingly challenging with an elevated genetic distance between target and reference or with the presence of contaminant sequences with high sequence similarity to the target species. The evaluation and testing of mapping efficiency and stringency are thus paramount for the reliable identification and analysis of ancient sequences. In this paper, we present ‘TAPAS’, (Testing of Alignment Parameters for Ancient Samples), a computational tool that enables the systematic testing of mapping tools for ancient data by simulating sequence data reflecting the properties of an ancient dataset and performing test runs using the mapping software and parameter settings of interest. We showcase TAPAS by using it to assess and improve mapping strategy for a degraded sample from a banded linsang (*Prionodon linsang*), for which no closely related reference is currently available. This enables a 1.8-fold increase of the number of mapped reads without sacrificing mapping specificity. The increase of mapped reads effectively reduces the need for additional sequencing, thus making more economical use of time, resources, and sample material.

## 1. Introduction

DNA retrieved from ancient or historical specimens is typically highly degraded into small fragments with damage-derived nucleotide mis-incorporations that complicate sequence analysis [1,2]. Furthermore, large amounts of contaminant molecules are often present in the sample, which can hamper the identification of endogenous DNA sequences [3]. The specific computational challenges posed by ancient DNA (aDNA) data were identified early on in the high-throughput sequencing era, which has led to a number of recommended tools and adjustments (e.g., [1,4,5,6,7,8]).

A commonly used approach for the analysis of ancient sequence data involves the local alignment or ‘mapping’ of sequence reads to a reference genome. Since the introduction of high-throughput sequencing, a large number of mapping tools have been developed with their own repertoire of parameters to fine-tune their performance (see [9]). This multitude of mapping tools as well as potential interactions between specific mapping parameters can make it difficult to select the most appropriate approach to maximize mapping performance for a specific dataset. A number of studies have addressed this problem by exploring and comparing the behavior of different mapping tools and their parameters [9,10,11,12]. However, very few studies have focused specifically on the challenges that are posed by aDNA data [1,13].

The inherent difficulties of mapping aDNA data are even more pronounced when only a distantly related reference is available [14,15]. In such cases, sequence divergence between the target species and reference can hamper successful mapping of the endogenous reads [16]. Most sequence read aligners (e.g., Burrows-Wheeler Aligner, BWA [17]; Bowtie [18]) allow the adjustment of alignment stringency. However, relaxing the allowed mismatches between sequence and target may also cause contaminant sequences to map to the reference. This issue is particularly problematic when working on species that are highly divergent from available reference species and/or species that are closely related to common contamination sources such as humans, cats, dogs, or pigs [19,20]. Various studies have shown that when reducing the stringency of mapping parameters, mapped contaminant reads can interfere with results of downstream analyses [3,21,22]. This illustrates the need for a tool that can systematically assess the effect of the mapping tool and its parameters (e.g., Teaser [10] Rabema [11], GCAT [23]). However, the distinctive challenges posed by aDNA data should be taken into account for mapping optimization, which may not be applicable for data derived from high quality DNA obtained when sampling living organisms.

**Figure 1:**
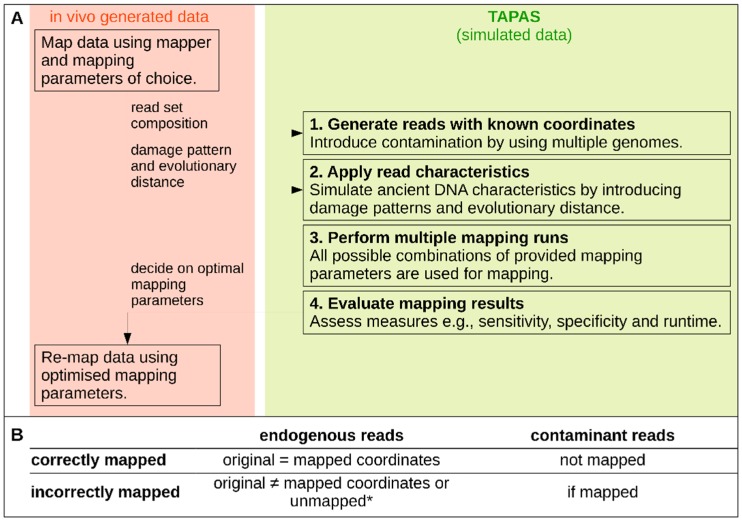
(**A**) Schematic of the TAPAS (Testing of Alignment Parameters for Ancient Samples) tool indicating a typical workflow from in vivo data to mapping assessment. (**B**) Different classes of reads that TAPAS assigns* incorrect and unmapped reads are by default not independently considered but can be distinguished if needed.

In this article we present TAPAS, which is a tool that allows the Testing of Alignment Parameters for Ancient Samples (see Figure 1). TAPAS achieves this by simulating sequence reads with user-defined characteristics such as the read length, sequence divergence, damage patterns, and contaminant sequences. TAPAS systematically tests a range of mapping parameters selected by the user. The output can then be investigated in terms of the sensitivity (fraction of all endogenous reads that map correctly to the reference genome), specificity (fraction of all contaminant reads that fail to map to the reference), and the false positive rate (mapped contaminant reads compared to the total number of mapped reads). In this way, TAPAS offers the opportunity to test the effects of multiple mapping parameter combinations on a simulated dataset representing the characteristics of any in vivo generated data. TAPAS allows the user to make an informed decision on which parameters to use for their data based on the desired mapping effectiveness and stringency.

## 2. Materials and Methods

### 2.1. The TAPAS Tool

The TAPAS tool is a pipeline of shell and python scripts designed to automate the assessment of mapping parameters and should run on any GNU/Linux environment. The only required pre-installed dependencies are R v ≥ 3.2, Python v ≥ 3.4 and the Python package manager ‘pip’. To improve the first-time usability and minimize the impact on the users' system, R and Python packages are loaded from a directory within TAPAS and can be downloaded and installed using a single command. TAPAS analyses conducted in this study utilized a Linux system running Scientific Linux v6.9, Python v3.5.0, and R v3.4.3. A schematic workflow is shown in Figure 1. A detailed manual and walkthrough also accompanies the TAPAS distribution (GitHub page: https://mlell.github.io/tapas).

#### 2.1.1. Data Simulation

The TAPAS tool is designed to simulate sequence reads with custom properties including average read length, damage patterns, contaminant sequences, and the sequence divergence to the reference. To this end, TAPAS can take any multi-FASTA file (i.e., a genome assembly comprising multiple scaffolds available from GenBank or other public repository) and simulate sequence read data in a standard format (i.e., FASTQ file). The read length distribution can be specified by the user. The desired patterns of nucleotide mis-incorporation in the simulated read set can be inferred from preliminary data exploration using the commonly-used tool mapDamage [24] or alternatively specified by the user. Simulated endogenous reads can be subjected to random base substitutions, which would reflect genetic distance between the target sequence and reference genome. Finally, using TAPAS, it is also possible to generate simulated metagenomic datasets comprising of sequences from both endogenous and contaminant (exogenous) sources at a ratio decided by the user. This allows for a direct assessment of the amount of false mapping of contaminant sequences. Modeling of fragment length distribution, genetic divergence, and DNA damage of contaminant sequences can be performed in the same way as described for endogenous sequences. The flexibility provided by TAPAS also allows the results of preliminary data analysis to be used to estimate appropriate parameter values for data simulation such that simulated datasets can be tailored precisely for specific research questions and objectives.

#### 2.1.2. Automated Read Mapping and Evaluation

TAPAS allows the automation of short read alignment for any combination of mapping parameters of interest. This allows users to directly and easily assess the effect of and potential interactions between a wide variety of parameter settings in a straightforward and reproducible fashion. For the work presented here, the commonly-used mapping software BWA v0.7.8 [17] has been used. Other mapping algorithms can also easily be employed for this step (details on how to implement other mapping tools are available in the documentation available from https://github.com/mlell/tapas). The mapping algorithm is integrated into the analysis pipeline by simply providing the respective program call to TAPAS. For studying the effect of a mapping parameter, the user can provide a placeholder in the call. The parameter values to be tested are then indicated in a configuration file based on which TAPAS performs mapping runs with all possible combinations of parameter values. The final output comprises a table reporting the results of all mapping runs.

For each mapping run, the sensitivity and specificity are reported. These two measures summarize the most important outcomes of read mapping analysis. Sensitivity represents the fraction of all endogenous reads that map correctly to the reference genome. For our purposes, unmapped and incorrectly mapped are both considered ‘incorrect’. Specificity represents the fraction of all contaminant reads that fail to map to the reference. For this purpose, the original genome coordinates are compared with the reported mapping coordinates to assess read mapping for each run. Endogenous reads can either be assigned as correctly mapped (positions match), incorrectly mapped (positions do not match), and unmapped. Contaminant reads are either incorrectly mapped (when the read is mapped) or correct (when the read is not mapped) (see Figure 1B). Based on the sensitivity, specificity, and runtime, the parameter combination which is most appropriate for the simulated dataset can then be used for the in vivo dataset of interest.

### 2.2. In Vivo Generated Linsang Dataset

To demonstrate the application of TAPAS, we used the tool to assess and optimize the mapping parameters for in vivo sequence data recovered from a banded linsang (*Prionodon linsang*). This sample was chosen based on two criteria. First, the data from this elusive carnivore was retrieved from a preserved skin sample, which is generally expected to yield highly degraded DNA [25]. Second, no intrageneric nuclear genome is available to serve as mapping reference for this species. At the time of writing, the closest available reference genome is the domestic cat (*Felis catus*), which is estimated to be around 28 million years diverged from the linsang [26]. The poor-quality sample and the expected sequence divergence between target species and reference are likely to result in suboptimal mapping results, which can be both assessed and optimized using TAPAS. Details on wet lab sample processing can be found in the Supplementary Material (File S1) [20,27,28,29,30,31,32]).

Using TAPAS, simulated endogenous sequence data was generated from the cat genome (*F. catus* v6.2, GenBank Accession number GCA_000181335.2) by applying 5% random nucleotide substitutions to approximate the expected evolutionary distance between cat and linsang. The composition of the simulated dataset (Table S1) was based on analysis of the in vivo dataset using FastQ Screen v0.5.0 (Supplementary Materials, Figure S1, Table S2). Exogenous (contaminant) reads were generated from the human genome (*Homo sapiens* GrCH38), which represents a likely source of contamination, and the dog genome (*Canis lupus familiaris* v3.1), which represents a closely related species to linsang that could potentially interfere with mapping results when the alignment mismatch parameters are relaxed. As bacterial and fungal DNA is generally the most abundant source of contamination [33,34], a total of five bacterial and fungal genomes were selected based on the BLAST results (Supplementary Material, Table S1).

For mapping using BWA *aln*, we chose to investigate the parameters defining the number of allowed mismatches between read and reference (*n*) and seed length (*l*) since these have previously been suggested to influence the mapping results [1,12,35]. Allowing more mismatches between read and reference is thought to result in increased sensitivity at the expense of decreased specificity [1]. However, it may allow an increase of false mapping of contaminant sequences. Disabling of the mapping seed function has previously been advocated for aDNA in order to utilize data for complete DNA fragments, which frequently contain an excess of nucleotide mis-incorporations at their terminal ends [36] and can be achieved by setting seed length to a value much greater than the expected fragment length. The range of values tested for *n* and *l* (Table S3) resulted in 30 individual mapping runs.

**Figure 2:**
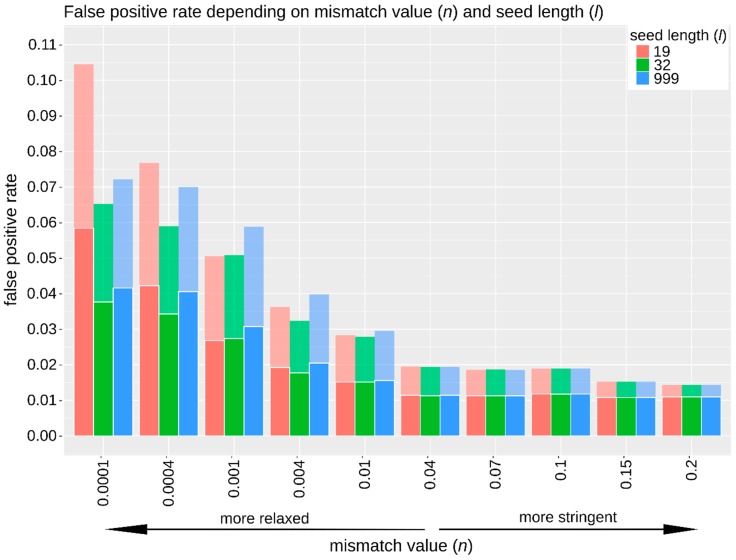
False positive rates calculated from all mapped reads (faded colors) and from all mapped reads with high mapping quality (MapQ > 30; dark colors). A total of 30 combinations of the parameters mismatch value (*n*, *x*-axis) and seed length (*l*, coloured bars, see key top right) were tested by using one million simulated reads and the cat genome as reference. Even at the most relaxed mismatch value tested, less than 6%of contaminant reads mapped successfully to the reference genome after quality filtering. This figure was generated using R (v3.4.2 and v3.4.3 [37]).

Mapping runs for simulated data were assessed based on their sensitivity, specificity, and runtime. Additionally, false positive rates (contaminant reads mapped compared to the total number of mapped reads) were investigated (Figure 2). After selecting TAPAS-optimized parameters, these parameters were used to re-map the in vivo generated data and compare their performance in terms of the percentage of mapped reads, read length, damage patterns, and coverage (Table 1, Figure S2). Finally, it should be noted that this example serves primarily to demonstrate the utility and application of the TAPAS tool in a real-world scenario rather than an exhaustive investigation of optimal mapping procedures for this particular organism. If biological conclusions were to be drawn from this dataset, then further testing of additional mapping references and contaminating organisms as well as additional mapping parameters and algorithms may be desirable and allow for further mapping optimization.

### 2.3. Published Bison Dataset

In order to showcase the TAPAS tool on a previously published dataset, we evaluated the mapping efficacy for recently published sequence data generated from a historical European bison sample (*Bison bonasus* [38]). We selected this dataset because it illustrates a relevant challenge for admixture studies where mapping to the genome of a potentially admixing closely related species (in this case the cow, *Bos taurus*) may bias admixture estimates, and thus requires the use of a more divergent mapping reference that is outgroup to the focal clade [38]. We followed the same procedure for this dataset as for the linsang dataset, using a subsample of 10 million reads as input in order to make the size of the bison dataset comparable to the linsang dataset (Supplementary Material, File S1 for details). Sequence reads representing endogenous data were generated from the water buffalo (*Bubalus bubalis*). Seven different organisms were selected as contaminant sequences (Table S1). Mapping was performed using the water buffalo (GenBank accession GCF_000471725.1) as a reference sequence. The estimated divergence time between water buffalo and bison (~11 M years, [39]) is more recent than that found for the domestic cat and linsang. Furthermore, a higher sequence similarity is expected because substitution rates are generally slower in larger bodied animals [40].

## 3. Results

### 3.1. In Vivo Linsang Data

For the in vivo linsang data, a total of 6,178,108 reads were available for mapping to the cat genome after adapter trimming. Mapping using BWA *aln* with default mapping parameters resulted in 580,773 mapped reads (9.40% of reads available after adapter trimming) and a coverage (number of covered bases divided by the size of the reference genome) of 0.01-fold. The endogenous content of the linsang sample was roughly estimated to be around 26% with some contaminants from common sources such as cow (~6%), dog (~13%), and human (~7%) (estimated using FastQ Screen; Figure S1). The majority of reads (about 70%) could not be mapped to any of the chosen reference sequences (Table S2), which could represent contaminant sources not included in the reference database. The mapped reads show damage patterns characteristic of archival DNA as well as a baseline substitution rate for all reads of about 3%, which is expected when mapping to a divergent reference and almost certainly represents an underestimate as more divergent reads may fail to map the data (Figure S2).

### 3.2. TAPAS

#### 3.2.1. Simulated Data Composition

Based on the preliminary results obtained using FastQ Screen, the simulated read set was generated to contain 26% cat sequences (to reflect the endogenous reads) and 74% contaminant reads (1% human, 3% dog, and 70% bacteria and fungi). Both endogenous and contaminant reads were then simulated following an exponential decay distribution as expected for degraded samples [2,41] with a minimum read length of 30 bp and a decay length of 15 bp (Supplementary Material, File S1). Additionally, following the mapDamage output from the in vivo data, random substitutions (5%) were introduced in the endogenous reads to reflect the sequence divergence between the cat and linsang as well as C to T substitutions to reflect aDNA damage. In total, one million reads were simulated as input for performing the mapping simulations.

#### 3.2.2. Sensitivity, Specificity, and False Positive Rate

**Figure 3:**
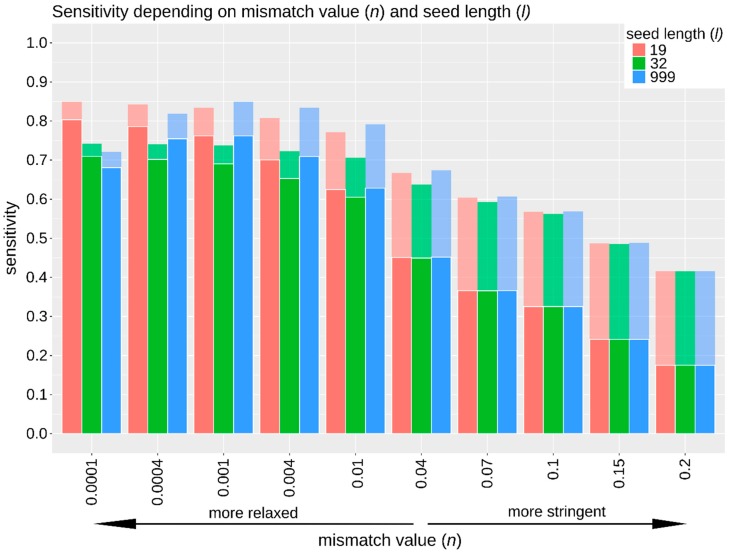
Sensitivity using BWA *aln* before (faded colors) and after (darker colors) filtering reads with low mapping quality (MapQ < 30). A total of 30 combinations of the parameters mismatch value (*n*, *x*-axis) and seed length (*l*, coloured bars, see key top right) were tested using one million simulated reads and the cat genome as reference. Increased sensitivity is achieved by relaxing the mismatch value. Furthermore, mismatch value and seed length appear to have an interactive effect where the impact of the seed length parameter on sensitivity is more pronounced at lower mismatch values. This figure was generated using R (v3.4.2 and v3.4.3, [37]).

The sensitivity when using different mapping parameters varied considerably, ranging from 18% to 80% of simulated endogenous reads that could be mapped correctly (Figure 3, Table S4). As expected, sensitivity increased with a more relaxed mismatch value (*n*) (up to 35% improvement compared to default settings) and decreased with more stringent mismatch values (up to 27% reduction compared to default settings). Our results also show that mismatch value and seed length may have a slight interactive effect where the optimal seed length differs with particular mismatch values (Figure 3). The general pattern remains unchanged before and after removing reads with low mapping quality (MapQ < 30), although more reads were lost for more stringent mismatch values (Figure 3). The mapping quality as assigned by BWA *aln* takes the sensitivity of the alignment algorithm into account, which leads to an overall reduction in mapping quality when a more stringent mapping approach is used (based on the source code from BWA). This resulted in a greater proportion of reads removed when applying a MapQ filter of 30 for more stringent mismatch values.

The specificity never dropped below 96% (Table S4). Furthermore, even at the most relaxed mismatch value (*n* value of 0.0001), the false positive rate was less than 6% (Figure 2). The number of incorrectly mapped endogenous reads never exceeded 0.23% for any mapping run (Table S4).

#### 3.2.3. TAPAS-Optimized Parameters

The simulated mapping results generated using TAPAS provide an approximation of the effect of different mapping parameters for the in vivo dataset. The optimal parameter combination was selected based on the sensitivity, specificity, and runtime. We selected a more relaxed mismatch value of 0.004 (i.e., allowing for more mismatches) and seed length of 32 bp as most appropriate for our dataset since it recovered the highest sensitivity (65%; Figure 3, Table S4) without considerable sacrifice of specificity (still above 99%; Table S4). Although our results suggest that a further relaxed mismatch value (*n* < 0.0001) is likely to improve the sensitivity even further, the increased runtime (up to 5.05 core processing unit (CPU) days for one million reads, Figure S3) may make this approach computationally unfeasible for larger datasets.

The mapping results for the simulated reads mapped with default parameters (*n* = 0.04, *l* = 32) and with TAPAS-optimized parameters (*n* = 0.004, *l* = 32) showed an increase by 1.5-fold in the number of mapped reads and the endogenous content (corresponding to an increase in sensitivity by 20%, Table S4) and of 1.5-fold in coverage (Table 1).

**Figure 4:**
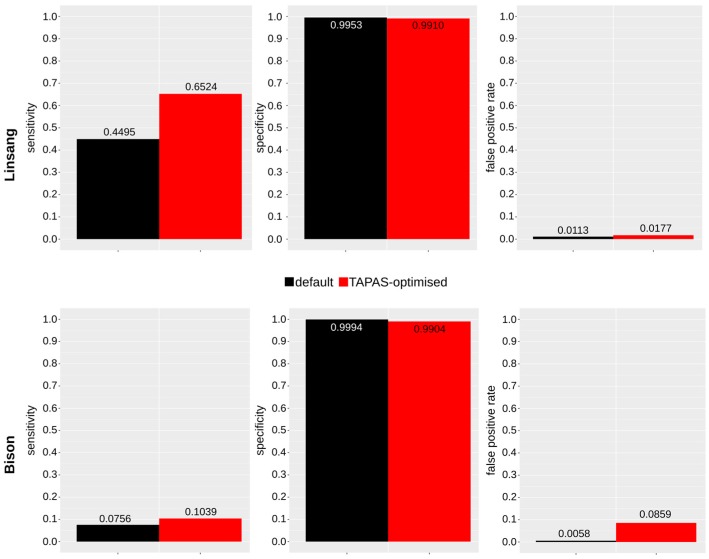
Sensitivity, specificity, and false positive rates of mapping using BWA *aln* with default parameters (black) and with the optimized parameters (red) based on the simulated data for the linsang (top) and bison (bottom). Using TAPAS, we can show an improvement of sensitivity (1.5-fold for the linsang and 1.4-fold for the bison) with only limited reduction in specificity while keeping the false positive rate low. This figure was generated using R (v3.4.2 and v3.4.3 [37]).

The TAPAS-optimized parameters (*n* = 0.004, *l* = 32) for mapping the in vivo data yielded a 1.8-fold increase of mapped reads compared to the default parameters and a 1.8-fold increase in coverage (Table 1, Figure 4). Assuming high specificity, the improvement of mapping results was therefore even higher than estimated based on the simulated reads.

### 3.3. Published Bison Data

Mapping the 10 million in vivo data subsample from the bison data with BWA *aln* by using the default mapping parameters resulted in 1.56 million mapped reads (15.6%, resulting in a coverage of 0.03-fold; Table S5). From the FastQ Screen results (Figure S1C), the data composition was estimated to be about 30% endogenous DNA with contaminants from human (~1%) and bacterial and fungal organisms (~69%) (Table S1). After performing read simulation and mapping evaluation using TAPAS (Supplementary Material, File S1), we choose values for the parameters *n* and *l*. With these settings, we achieved the highest sensitivity while still maintaining a specificity >99% (*n* = 0.0004, *l* = 19). We achieved a 1.4-fold improvement of sensitivity and a 1.4-fold increase in coverage (Figure 4, Tables S5 and S6).

## 4. Discussion

In this study, we show that the simulation of aDNA data sequence data is a viable and effective approach for the systematic optimization of mapping algorithms and parameters. Applying this approach to the linsang dataset yielded a 1.8-fold improvement in sequence recovery with no apparent sacrifice of specificity. We have furthermore applied the TAPAS tool to assess the mapping efficacy for a previously published dataset (European bison, [38]). We showed that, unlike the linsang dataset, only small improvements were possible for the bison dataset compared to the default parameters used previously. Potential reasons why this could be the case may be the properties of the reference genome [12], the reduced evolutionary distance between target and reference, or the longer fragment length. This confirms that different datasets behave differently and therefore are likely to benefit from individual mapping parameter assessment and optimization, which further underlines the value of the TAPAS tool for the structural assessment of mapping efficacy for ancient DNA datasets.

In line with previous studies [13], our analysis showed that BWA *aln* retains a high level of specificity when using extremely relaxed mismatch values even with a high abundance of contaminant sequences including those from closely related species. A commonly used mapping strategy in aDNA research [36,42] is to systematically disable the seeding when mapping aDNA with BWA *aln* [1]. However, both reducing the seed length and disabling seeding almost always resulted in higher sensitivity values than the default seed length (*n* = 32). The effect of the seed length does become more pronounced as the mismatch value is relaxed, which indicates a potential interactive effect between these parameters. Such interactions are likely to complicate the optimization of mapping parameters and further support the benefit of the TAPAS tool for this task.

Although tools designed for datasets derived from high quality DNA are available (e.g., Teaser [10], Rabema [11], GCAT [23]), the characteristics of aDNA present specific challenges that are not common for modern genomic data. Issues such as spurious mapping of contaminants can potentially have a detrimental impact on mapping and subsequent analysis of ancient data and therefore most tools for benchmarking and optimization for standard datasets are less suitable for aDNA datasets. To date, only a single tool has been developed for simulating the specific characteristics of aDNA data (‘Gargammel’; [13]). Gargammel offers comprehensive aDNA data simulation including fragment size, base mis-incorporation patterns, and nucleotide composition to reflect the biases introduced by different sample types, library preparation methods, and sequencing protocols. Simulated data are then mapped to a reference following standard recommendations for processing aDNA data (following [1]). In contrast to Gargammel, TAPAS is specifically focused on identifying optimized mapping parameters in order to maximize the amount of usable data that can be generated from one sample while also giving an estimation of specificity and false positive rate. By recording the original coordinates during read generation, TAPAS allows for a clear distinction of correctly and incorrectly mapped and unmapped reads rather than determining only the change in the number of mapped reads. As such, it offers a more detailed evaluation of the mapping efficiency.

A further core component of TAPAS is the possibility to introduce a fixed genome wide mutation rate during read generation. While this is currently limited to a simplified representation of the evolutionary divergence between target species and the available reference genome, it does enable a structural evaluation of a more relaxed mismatch strategy for read alignment. This feature could be further improved by the implementation of specific nucleotide substitution models to mutate the sequences. Further refinements to data simulation could be achieved by introducing more complex models for generating read length distributions and patterns of nucleotide mis-incorporation as well as the simulation of short indels in the sequence reads. This would allow a more accurate representation of empirical datasets. An additional feature that could be added to future versions is a more detailed report of false positive mapping. For example, the distribution of falsely mapped reads across the genome and GC content could be used. TAPAS’ modular design and simple data formats should allow for a relatively straightforward implementation of any such refinements in the future. Although simulated datasets will never encompass the entire complexity of an empirical dataset, it nevertheless allows for a more objective and structural assessment of the alignment efficiency when mapping reads from a particular dataset.

## 5. Conclusions

Our study highlights the potential benefits of systematically optimizing mapping tools and their parameters when analyzing aDNA datasets. The effects of altering mapping parameter values and the interaction between parameters, may not be easy to predict and likely depend on a multitude of factors such as fragment size, the reference genome, and contamination. The TAPAS tool presented here provides a way of exploring these processes in an automated and reproducible manner. Furthermore, this study reinforces the potential for bioinformatic optimization in advancing the study of aDNA, which is likely to become increasingly important as future developments in wet lab procedures extend both the time depth and taxonomic breadth of sequence retrieval.

## Figures and Tables

**Table 1:** Summary of mapping results for in vivo generated data mapped with two sets of mapping parameters in comparison to mapping runs with simulated data applying the same mapping parameters for the linsang data.

	*n*	*l*	Reads for Mapping	Duplicates (%)	Reads Mapped	Reads Mapped (%)	Mapped bp	Coverage	Median Read Length	Total CPU Time (h)
	fold increase									
in vivo generated data	0.04 *	32 *	6,178,108	1.92	580,773	9.40	26,240,720	0.011	42	9.23
	0.004	32 *	6,178,108	2.06	1,017,849	16.48	46,032,861	0.019	42	33.50
				1.1	1.8	1.8	1.8	1.8	1.0	3.6
simulated data	0.04 *	32 *	1,000,000	0.78	117,379	11.74	5,802,202	0.002	44	0.10
	0.004	32 *	1,000,000	1.08	171,099	17.11	8,567,055	0.003	44	1.32
				1.4	1.5	1.5	1.5	1.5	1.0	13.5

*n*: mismatch value; *l*: seed length; * default mapping parameters of Burrows-Wheeler Aligner (BWA) *aln*; CPU: core processing unit.

## Data Availability

Linsang test data has been made available on the European Nucleotide Archive (ENA) under Study Accession number PRJEB25526.

## Supplementary Materials

The following are available online at http://www.mdpi.com/2073-4425/9/3/157/s1. File S1: Detailed experimental procedures for wetlab protocols and bioinformatic pipelines. Figure S1: FastQ Screen results for the in vivo generated dataset and the simulated dataset of the linsang and the bison sample: (A) linsang—in vivo generated dataset, (B) linsang—simulated dataset, (C) bison—in vivo generated dataset, (D) bison—simulated dataset. The majority of reads could not be matched to any of the genomes included in the database (Table S2). Figure S2: Extracts from mapDamage output for in vivo generated data and simulated data of the linsang sample using either default or TAPAS-optimized mapping parameters: (A) in vivo generated data mapped with default settings, (B) in vivo generated data mapped with TAPAS-optimized settings, (C) simulated data mapped with default settings, (D) simulated data mapped with TAPAS-optimized settings. Figures adapted from the output of mapDamage2 [9]. Graph colors represent C to T substitutions (red), G to A substitutions (blue), all other substitutions (grey), soft-clipped bases (orange), and deletions/insertions relative to the reference (green/purple). Both simulated and in vivo generated datasets show damage patterns characteristic of degraded DNA. Figure S3: Runtime in seconds (CPU time, *y*-axis) using BWA *aln* for mapping one million simulated linsang reads to the cat genome testing 30 combinations of the parameters mismatch value (*n*, *x*-axis) and seed length (*l*, colored bars, see key top right). Particularly at long seed lengths runtime becomes increasingly unfeasible for large datasets at more relaxed mismatch values. Bars for the longest runtimes are broken to aid visualization of shorter runtimes. This figure was generated using R (v3.4.2 and v3.4.3 [11]). Table S1: Genomes used to assemble the simulated datasets. Fractions of the endogenous and contaminant sequences were estimated from the in vivo data (described in Materials and Methods in main text). Table S2: Genomes included in the database for screening in vivo generated and simulated reads with FastQ Screen. Table S3: Values of mapping parameters systematically tested with TAPAS. The default settings of BWA *aln* are indicated with an asterisk. Table S4: Summary of the total number of endogenous (endo) and contaminant (exo) reads mapped correctly and incorrectly filtered for mapping quality (≥30; <30) for 30 mapping runs with BWA *aln* using 30 different combinations of the mapping parameters mismatch value (*n*) and seed length (*l*) for the simulated linsang data. Table S5: Summary of mapping results for in vivo generated data mapped with two sets of mapping parameters in comparison to mapping runs with simulated data applying the same mapping parameters for the bison data. *n* = mismatch value, *l* = seed length, * default mapping parameters of BWA *aln*, ⁺ random subsample of ten million reads. Table S6: Summary of the total number of endogenous (endo) and contaminant (exo) reads mapped correctly and incorrectly filtered for mapping quality (≥30; <30) for 30 mapping runs with BWA *aln* using 30 different combinations of the mapping parameters mismatch value (*n*) and seed length (*l*) for the simulated bison data.
