# Supplementary material for: Testing of Alignment Parameters for Ancient Samples: Evaluating and Optimizing Mapping Parameters for Ancient Samples Using the TAPAS Tool
Source: Genes (Basel). 2018 Mar 13;9(3):157. doi: 10.3390/genes9030157 (PMC5867878; doi:10.3390/genes9030157)
Supplement: Supplementary file 1 [file genes-09-00157-s001.zip › TAPAS_FigureS1_FastQScreen_12Mar2018.pdf]

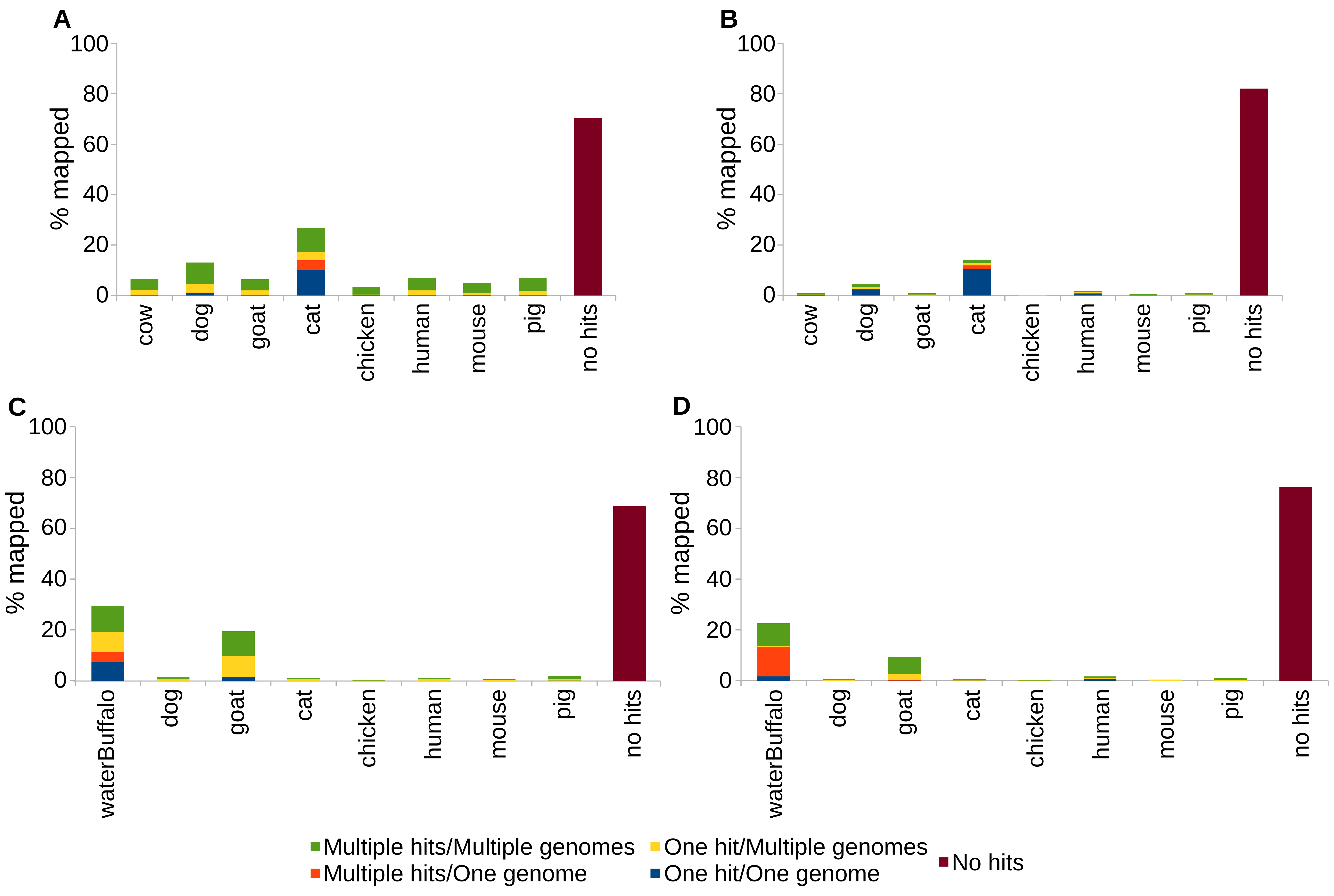
A
B
C
D
100
80
60
40
20
0
% mapped
cow
dog
goat
cat
chicken
human
mouse
pig
no hits
waterBuffalo
Multiple hits/Multiple genomes
One hit/Multiple genomes
Multiple hits/One genome
One hit/One genome
No hits
