# Supplementary material for: Testing of Alignment Parameters for Ancient Samples: Evaluating and Optimizing Mapping Parameters for Ancient Samples Using the TAPAS Tool
Source: Genes (Basel). 2018 Mar 13;9(3):157. doi: 10.3390/genes9030157 (PMC5867878; doi:10.3390/genes9030157)
Supplement: Supplementary file 1 [file genes-09-00157-s001.zip › TAPAS_FileS1_12Mar2018.pdf]

Supplementary Material for:

Testing of Alignment Parameters for Ancient Samples: Evaluating and optimising mapping parameters for ancient samples using the TAPAS tool

Ulrike H Taron, Moritz Lell, Axel Barlow and Johanna L A Paijmans

DNA extraction and library preparation

All lab work was performed in dedicated clean lab facilities at the University of Potsdam with appropriate contamination precautions in place [1]. DNA was extracted from 8.4 mg of tissue from a preserved skin. The digestion was performed using a buffer from Rohland et al. [2], consisting of 5M of GuSCN, 25 nM NaCl, 50 mM of Tris, 20 mM of EDTA, 1% Tween-20 and 1% betamercaptoethanol. After digestion, the protocol from Dabney et al. [3] was followed for binding and washing steps. From the resulting DNA extract, 10 µl were used to prepare single-stranded DNA libraries [4]. Approximately ten million 75 bp single-end reads were generated using the Illumina NextSeq 500 sequencing platform, using a custom Read 1 and Index Read 2 sequencing primers as needed for single-stranded libraries [4,5].

Bioinformatic procedures

The endogenous content, average fragment length, damage patterns (elevated C to T substitution levels at read ends) and potential contamination sources were estimated by mapping the linsang sequence data to the closest available nuclear genome; the domestic cat (*Felis catus* v6.2; GCA_000181335.2). The bioinformatic procedures in brief: first, adapters were removed, and reads shorter than 30bp were discarded using cutadapt v1.10 [6]. For read mapping and parsing, filtering reads with low mapping quality (MAPQ < 30) and removal of duplicates, BWA v0.7.8 and samtools v0.1.19 were used with default settings [7,8]. Damage patterns of the mapped reads were recovered using mapDamage v2.0.7 [9]. FastQ Screen v0.5.0 (available from: https://www.bioinformatics.babraham.ac.uk/projects/fastq_screen/) was utilised to estimate the relative proportions of vertebrates contaminant sequences present in the sample, by comparing the reads to a range of potential sources (Table S2, Figure S1A). To identify suitable bacterial and fungal contaminant sources, one million reads from the in vivo data were blasted using blastn against the full nucleotide database (downloaded from NCBI on December 4th, 2017). From the five most occurring bacterial and fungal genera, one genome was selected for each (Table S1).

Commands used for the linsang test case

This section describes the commands (TAPAS and standard UNIX commands) used to run the linsang test case. The commands were executed in a bash shell on a Linux system running Scientific Linux v6.9. More detailed descriptions can be found in the manual, which can be found at https://mlell.github.io/tapas .

1) Simulate sequence data from desired reference genomes

1.1. Create reads from cat genome (Table S1) to reflect endogenous sequences (260,000 reads, with a minimum read length of 30bp, and a decay length of 15bp)

```
uniform Felis_catus_6.2_all-dna-chromosomes.fasta \
  --seed 1234 \
  --output cat.coord cat.nucl \
  260000 30 15
```

1.2. Create unique read names

```
index-column --prefix cat_ \
  --colname name \
  --inplace cat.coord
```

1.3. Create a FASTQ file by combining sequences from cat.nucl and read names from cat.coord and adding a line with quality scores (quality scores are all ‘F’, a constant high value in the Phred+33 alphabet used by BWA to denote per-base quality)

```
synth-fastq cat.nucl \
  <(sed 's/./F/g' cat.nucl) \
  <(awk '(NR!=1){print $1}' cat.coord) \
  >cat.fastq
```

1.4. Create reads from human genome (Table S1) to reflect common contaminant source (in this case, 10,000 reads, with a minimum read length of 30bp, and a decay length of 15bp)

```
uniform homo.fasta \
  --seed 1567 \
  --output human.coord human.nucl \
  10000 30 15
```

1.5. Create unique read names and a FASTQ file for the human reads according to the cat example (see 1.2 - 1.3)

1.6. Create reads from dog genome (Table S1) to reflect contaminant with high sequence similarity to target species (in this case, 30,000 reads, with a minimum read length of 30bp, and a decay length of 15bp)

```
uniform CanFam3.1_all-dna-chromosomes.fasta \
  --seed 1394 \
  --output dog.coord dog.nucl \
  30000 30 15
```

1.7. Create unique read names and a FASTQ file for the dog reads according to the cat example (see 1.2 - 1.3)

1.8 Create reads from five bacterial and fungal genomes (Table S1) to reflect contaminants (in this case, 700,000 reads in total, i.e. 140,000 reads per contaminant genome, with a minimum read length of 30bp, and a decay length of 15bp and a unique seed for each contaminant species). An example for one of the contaminant genomes is given below.

```
    uniform GCF_001457475.1_NCTC10807_genomic.fna \
     --seed 2201 \
     --output A.xylo.coord A.xylo.nucl \
  140000 30 15
```

1.9. Create unique read names and a FASTQ file for all bacterial and fungal reads following the same procedure as described for the cat genome (see 1.2 - 1.3)

2) Introduce damage pattern and simulate evolutionary distance for the 'endogenous' sequences

2.1 Extract damage patterns from nucleotide misincorporation output files (*_freq.txt) generated by mapDamage [9], based on the in vivo data.

```
    mapdamage2geomparam \
     --fit-plots fit_ \
     *_freq.txt \
     > mut.tbl
```

2.2. The resulting output file (mut.tbl) can then be modified to reflect both specific nucleotide misincorporations to reflect ancient DNA damage, as well as an estimated sequence divergence to reflect evolutionary distance. For specific nucleotide changes the intercept was set to zero. Furthermore, a third line of code was added to account for 5% sequence divergence to reflect the estimated evolutionary distance between reads and reference, for which no specific base changes, a factor and geometric probability of zero and an intercept of 0.05 were specified.

```
    awk 'Begin {OFS="\t"} (NR!=1){$6="0"}{print} END {print
  "3","*","*","0","0","0.05"}' mut.tbl > mut.tbl.2
```

The file mut.tbl.2 after the modifications described above.

```
    column -t mut.tbl.2
    strand  from  to  factor   geom_prob  intercept
    3p      C     T   0.1048   0.721      0
    5p      C     T   0.03675  0.953      0
    3p      *     *   0        0          0.05
```

2.3. Apply the damage pattern and evolutionary distance to the endogenous reads

```
 filter-fastq --nucleotide @ \
   multiple_mutate mut.tbl.2 @ \
   < cat.fastq \
   > cat_mut.fastq
```

3) Combine all reads to one FASTQ file

```
    cat A.ory.fastq \
    A.xylo.fastq \
    G.lozo.fastq \
    P.chry.fastq \
    R.etli.fastq \
    cat_mut.fastq \
```

```
        dog.fastq \
        human.fastq \
   > all.fastq
```

4) Prepare parameter values for mapping parameters

4.1. In this case two separate files n.par and l.par holding all values for each parameter listed in Table S3 were created using a standard text editor (e.g. nano).

4.2. Create a table containing all possible combination of chosen mapping parameter values and add an index column to the table.

```
        cross_tab --head 1 *.par > partab
        index-column --colname runidx --inplace partab
```

5) Execute mapping runs

5.1. Create mapping calls.

```
        table2calls partab map-bwa_aln.sh \
          > calls
```

Script map-bwa_aln.sh used for the linsang test case.

```
        #!/bin/bash
        ## This script performs a mapping using BWA.
        ## It requires the variables l, n, runidx be set
        ## prior to its execution.

        # Fail if any needed variable is not set
        set -ue

        bwa aln -n ${n} -l ${l}      \
        /folder/Felis_catus_6.2_all-dna-chromosomes.fasta \
        /folder/all.fastq \
        > /folder/${runidx}.sai   \
        2> /folder/${runidx}.log   &&

        bwa samse                    \
    /folder/Felis_catus_6.2_all-dna-chromosomes.fasta \
    /folder/${runidx}.sai     \
    /folder/all.fastq         \
    > /folder/${runidx}.sam   \
    2>> /folder/${runidx}.log

        rm /folder/${runidx}.sai
```

5.2. Start execution of mapping runs.

```
        mcall -c calls -t 10 --status
```

6) Parse sam files

6.1. Extract prefix on sam files. The content of the for-loop opened here continues until step 6.8.

```
        for sam in /folder/*.sam; do
      bn=$(basename $sam)
```

```
p=${bn%.sam}
```

6.2. Convert sam files into tables for better parsing. Extracting information on query name (qname), reference name (rname), mapping position (pos) and mapping quality (mapq).

```
sam-extract \
--sam-fields qname,rname,pos,mapq \
${p}.sam > ${p}.tab
```

6.3. Add additional columns “true_organism” and “mapped_organism” to the table, which contain information on the organism the reads originate from (true_organism) and the reference genome the reads have been mapped to (mapped_organism).

```
add_mapped_organisms \
--endogenous cat Felis_catus_6.2_all-dna-chromosomes.fasta.fai cat.coord \
--exogenous dog CanFam3.1_all-dna-chromosomes.fasta.fai dog.coord \
--exogenous bacteriafungi GCF_000184455.2_ASM18445v3_genomic.fna.fai
A.ory.coord \
--exogenous bacteriafungi GCF_001457475.1_NCTC10807_genomic.fna.fai
A.xylo.coord \
--exogenous bacteriafungi GCF_000409485.1_GLAREA_genomic.fna.fai
G.lozo.coord \
--exogenous bacteriafungi GCA_000710275.1_ASM71027v1_genomic.fna.fai
P.chry.coord \
--exogenous bacteriafungi Rhizobium_etli_cfn_42.ASM9204v1.dna.toplevel.fa.fai
R.etli.coord \
--exogenous human homo.fasta.fai human.coord \
${p}.tab > ${p}.mod.tab
```

6.5. Check if reads have been mapped correctly by comparing the original genome coordinates with the reported mapping coordinates. In this test case a read is considered correctly mapped if it was mapped to the exact same position it has been extracted from.

```
pocketR 'within(input, {
  correct =
         mapped_pos == true_start &
         mapped_rname == true_record &
         mapped_organism == true_organism})
  '  ${p}.mod.tab > ${p}.TrueFalse.tab
```

6.6. Grouping of reads into a single file, which simplifies generating plots in R.

```
pocketR '
aggregate( cbind(count=qname)
    ~ true_organism + mapped_organism + correct,
      FUN=length, data=input)
' ${p}.TrueFalse.tab > ${p}.agg
```

6.7. Plot the read fate of a single mapping run. The output are bar plots showing the fractions of unmapped reads and reads mapped to the reference, for each organism reads have been created from.

```
plot-read-fate true_organism mapped_organism \
  correct count ${p}.pdf ${p}.agg
```

6.8. Extract measures sensitivity and specificity

```
sensspec --c-morg mapped_organism \
  --c-torg true_organism \
```

```
    ${p}.agg cat \
    > ${p}.parameters

  echo "$sam done. -> Generated ${p}.{tab,agg,pdf,performance}"

  done  # end of for loop (step 6.1)
```

7) Compare multiple mapping runs

7.1. Add the run index to each parameter file. The result is a single-line table for each mapping run with measures for sensitivity and specificity connected to the run index of each mapping run.

```
for f in /folder/*.parameters; do
        i=$(basename ${f%.parameters})
        add_const_column "$f" runidx "$i" > ${i}.performance
done
```

7.2. Concatenate all performance files. The result is a multi-line table containing measures for sensitivity and specificity connected to the run index of each mapping run, one line per mapping run.

```
cat_tables *.performance > performance
```

7.3. Connect mapping parameters with the results of the mapping runs. Based on the run index, sensitivity and specificity for each mapping run can be related to the used parameter combination.

```
merge -a performance runidx \
        -b partab runidx \
        --all-a-cols --all-b-cols --all-a \
        | write_later performance
```

8) Apply a quality filter (mapq 30) to the mapping results

```
  for f in /folder/*.sam; do
i=$(basename ${f%.sam})
samtools view -Sh -q 30 ${i}.sam > ${i}_filtered.sam
  done
```

9) Parse filtered sam files according to steps 6) and 7)

European bison sample

Bioinformatic procedures

Data from a historical European bison sample (Cc1/8853; *Bison bonasus*) was utilised to showcase the use of TAPAS on a previously published dataset. Details on sample processing and sequencing can be found in the original publication [10]. Raw data was downloaded from the SRA (Accession number SRR4996860). Cutadapt v1.12 was used to trim reads and discard reads shorter than 30 bp [6]. After adapter trimming, a subsample of ten million random reads was extracted using seqtk v1.2 (available from: https://github.com/lh3/seqtk). Reads were mapped to the genome of the water buffalo (*Bubalus bubalis*, GCF_000471725.1), following the same procedure as described for the linsang data.

Based on the results from the first mapping run (with default parameters), simulated endogenous and contaminant reads were generated using the TAPAS pipeline. Reads were simulated from the selected FASTA files (Table S1) with the uniform script from TAPAS, using a minimum length of 30 bp and a decay length of 200 bp (discarding reads >75 bp). For one of the simulated read sets, an example is described below.

```
### Simulate reads with a length between 30 and 75 bp
### Output: tab-separated table holding information on the read location (record, start, end) and the read sequence
        uniform Pseudomonas_putida_KT2440_chromosome.fasta --seed 1987 850,000 30 200 | awk "(length(\$4) < 75)" > P.puti.tab

### Extract the exact number of reads (accounting for one header line in the *.tab file)
        head -n 115001 P.puti.tab > P.puti_short.tab

### Generate the files *.nucl and *.coord
        awk 'BEGIN{OFS="\t"}{print $1,$2,$3}' < P.puti_short.tab > P.puti.coord
        awk '{print $4}' P.puti_short.tab | tail -n+2 > P.puti.nucl

### Continue with the TAPAS pipeline from step 1.2 “Create uniq read names”
```

In total, one million reads were simulated for mapping evaluation (Table S1). To approximate the expected sequence divergence between the bison sample and the water buffalo reference, random base substitutions (4%) were introduced in the endogenous reads, as well as C to T substitutions at the 3’ and 5’ end of the reads, to reflect aDNA damage. All other steps were executed exactly as described for the linsang dataset.

Figures

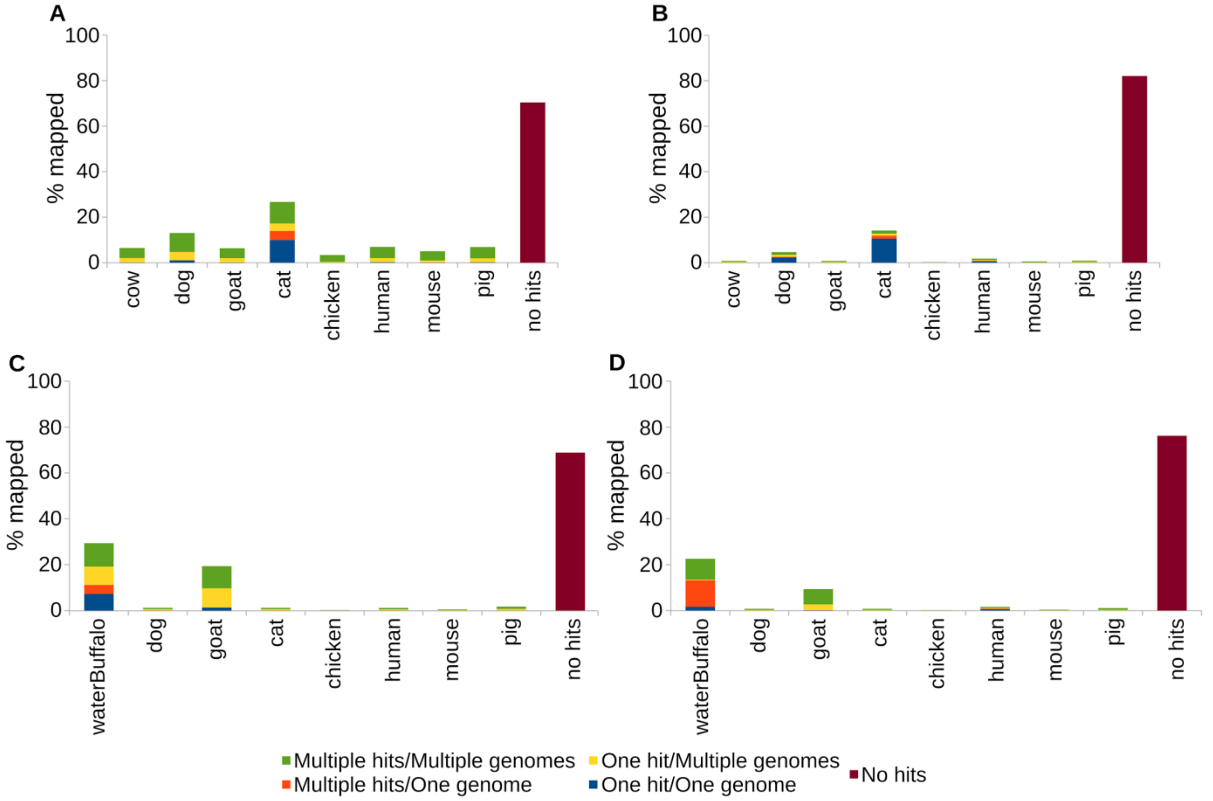

Figure S1. FastQ Screen results for the in vivo generated dataset and the simulated dataset of the linsang and the bison sample: (A) linsang - in vivo generated dataset, (B) linsang - simulated dataset, (C) bison - in vivo generated dataset, (D) bison - simulated dataset. The majority of reads could not be matched to any of the genomes included in the database (Table S2).

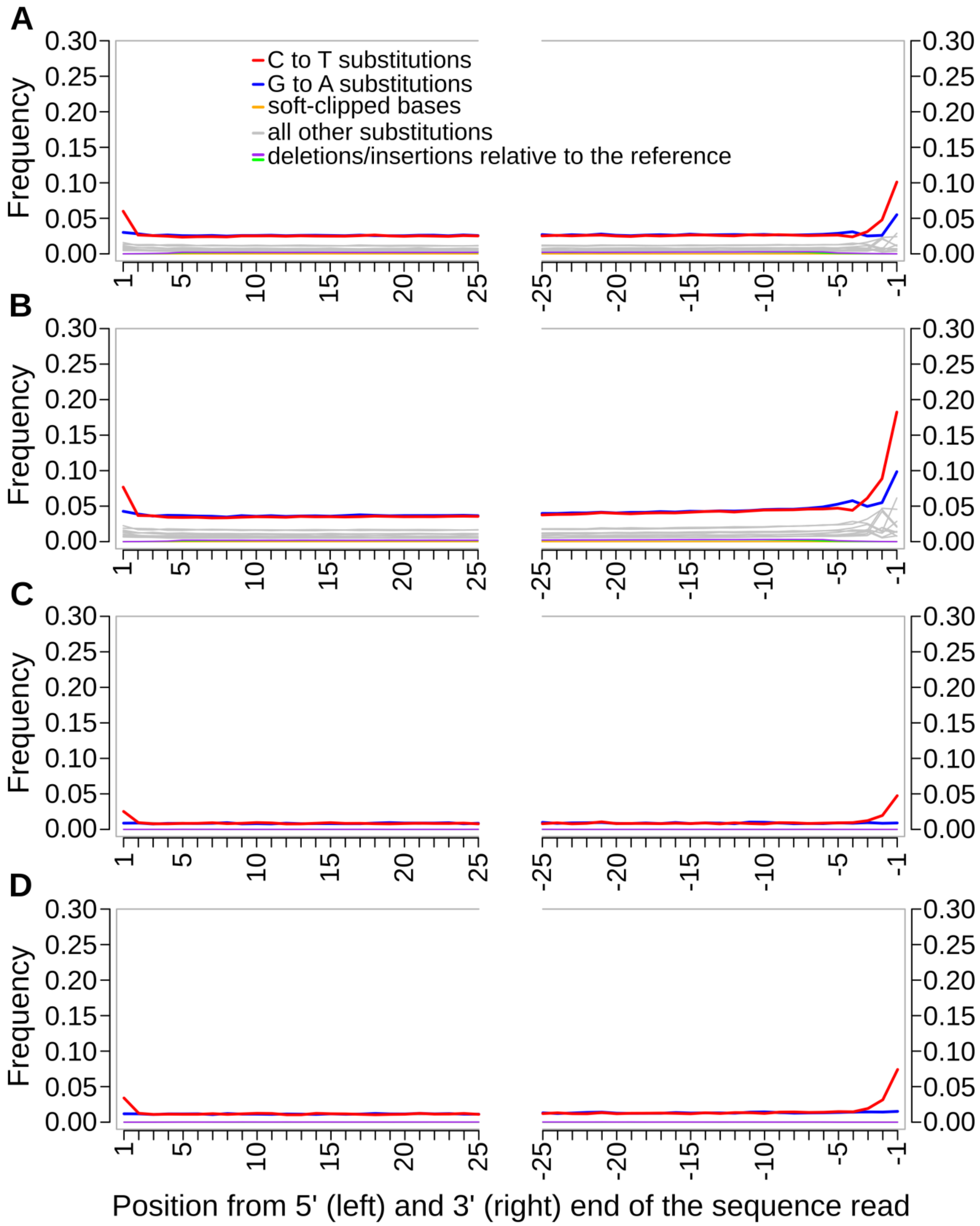

Figure S2. Extracts from mapDamage output for in vivo generated data and simulated data of the linsang sample using either default or TAPAS-optimised mapping parameters: (A) in vivo generated data mapped with default settings, (B) in vivo generated data mapped with TAPAS-optimised settings, (C) simulated data mapped with default settings, (D) simulated data mapped with TAPAS-optimised settings. Figures adapted from the output of mapDamage2 [9]. Graph colours represent C to T substitutions (red), G to A substitutions (blue), all other substitutions (grey), soft-clipped bases (orange), and deletions/insertions relative to the reference (green/purple). Both simulated and in vivo generated datasets show damage patterns characteristic of degraded DNA.

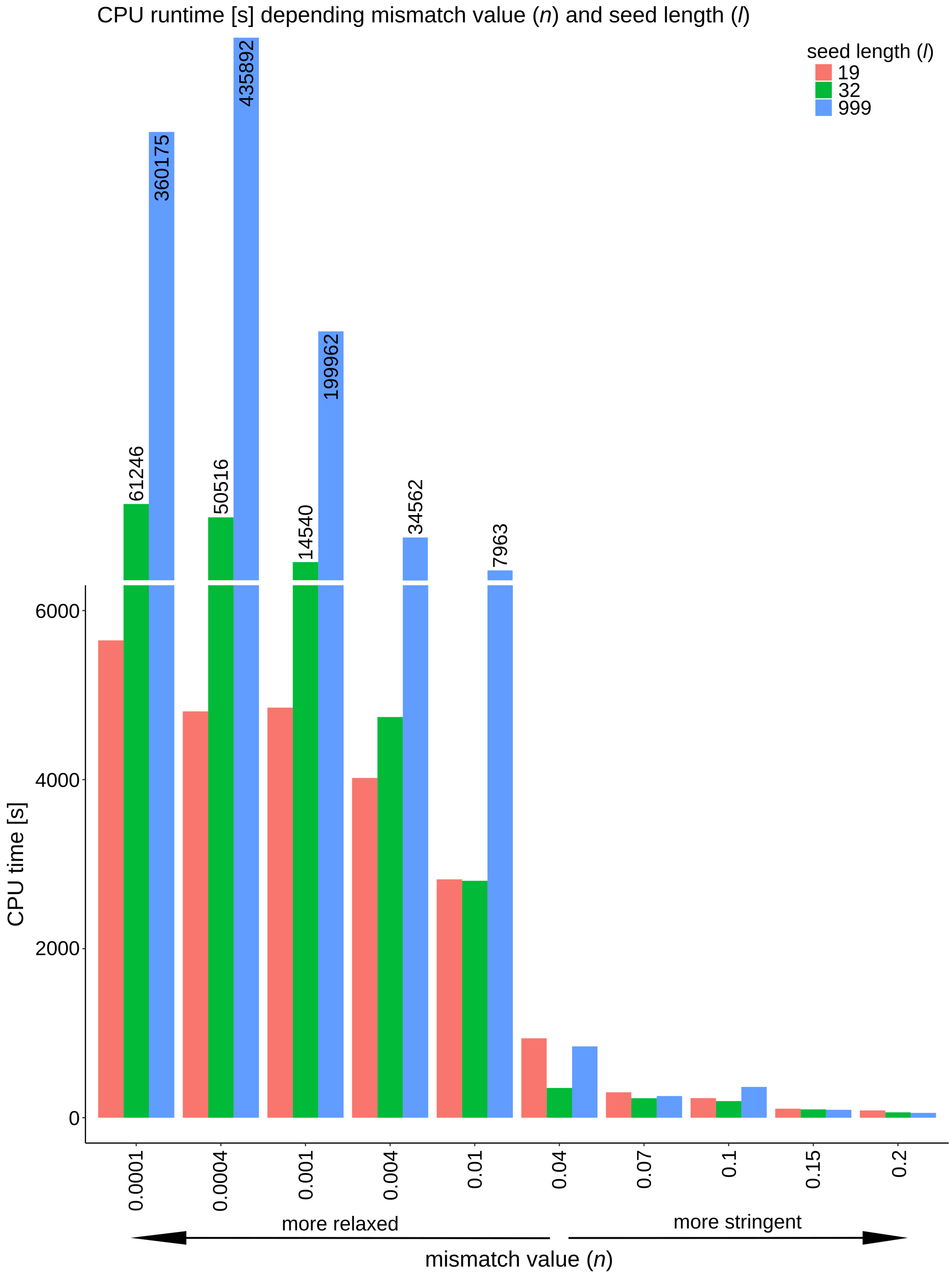

Figure S3. Runtime in seconds (CPU time, y-axis) using BWA aln for mapping one million simulated linsang reads to the cat genome testing 30 combinations of the parameters mismatch value (n, x-axis) and seed length (l, coloured bars, see key top right). Particularly at long seed lengths runtime becomes increasingly unfeasible for large datasets at more relaxed

mismatch values. Bars for the longest runtimes are broken to aid visualisation of shorter runtimes. This figure was generated using R (v3.4.2 and v3.4.3 [11]).

Code used to create Table S4 and S6

Disclaimer:

This file contains the code used for generating tables and figures presented in the TAPAS manuscript. These are custom scripts (using basic linux command line utilities and R) suited for a particular folder and file structure, hence paths and file names have been hard coded. The code is not optimised for a "copy and use" approach in a different environment, but rather to present the code underlying the figures in the TAPAS manuscript. The code is free to be adapted and re-used.

Input: *.TrueFalse.tab
Output: tab-separated file containing raw data for all categories of reads (Table S4 and S6) from each mapping run (one row per run).
Notes: The header can be added manually by concatenating a file holding only the header (numbers_from_tab_HEADER.txt) and the file "number_from_tab.txt" resulting in the file "numbers_from_tab_w_HEADER.txt".

```
### Pipeline to parse multiple tab files resulting from different mapping runs with different parameters and
one read set.
### Pipeline is adapted to my folder structure NEEDS to be modified when applying it to different data.

#!/bin/bash
set -ue

### Script to extract more detailed numbers from ".TrueFalse.tab files
for f in /path/to/input/*.TrueFalse.tab; do
	i=$(basename ${f%.TrueFalse.tab})
# echo $i

### number of endogenous reads (assuming cat is endogenous and everything else is exogenous)
### true_organism must be cat
# echo "number of endogenous reads (assuming cat is endogenous and everything else is exogenous). Should
be 260000"
En=$(awk '($9=="cat"){print $0}' ${i}.TrueFalse.tab | wc -l)

### number of exogenous reads
 ### true organism must not be cat and not true_organism (=header)
# echo "number of exogenous reads. Should be 740000"
Ex=$(awk '($9!="cat" && $9!="true_organism"){print $0}' ${i}.TrueFalse.tab | wc -l)

### endogenous reads
######################################################################
##

### number of endogenous reads mapped w min mapq 30
# echo "number of endogenous reads mapped w min mapq 30"
# $9=="cat": reads are endogenous
# $4>=30: mapping quality over 30
Ena30=$(awk '($9=="cat" && $4>=30){print $0}' ${i}.TrueFalse.tab | wc -l)
```

```
### number of endogenous reads correctly mapped w min mapq 30
# echo "number of endogenous reads correctly mapped w min mapq 30"
# $9=="cat": reads are endogenous
# $4>=30: mapping quality over 30
# $10=="TRUE": reads are mapped correctly (true_organism=mapped_organism &&
true_record=mapped_rname && true_start=mapped_pos)
Ena30T=$(awk '($9=="cat" && $4>=30 && $10=="TRUE"){print $0}' ${i}.TrueFalse.tab | wc -l)

### number of endogenous reads incorrectly mapped w min mapq 30
# echo "number of endogenous reads incorrectly mapped w min mapq 30"
# $9=="cat": reads are endogenous
# $4>=30: mapping quality over 30
# $10==""FASLE": reads are mapped incorrectly (true_organism!=mapped_organism and/or true_record!
=mapped_rname and/or true_start!=maooed_pos)
# $5!="*": only mapped reads
Ena30F=$(awk '($9=="cat" && $4>=30 && $10=="FALSE" && $5!="*"){print $0}' ${i}.TrueFalse.tab | wc -l)

### number of endogenous reads mapped w mapq < 30
### This number also includes unmapped endogenous reads
# echo "number of endogenous reads mapped w mapq < 30"
# $9=="cat": reads are endogenous
# $4<30: mapping quality below 30
Enb30=$(awk '($9=="cat" && $4<30){print $0}' ${i}.TrueFalse.tab | wc -l)

### number of endogenous reads incorrectly mapped w mapq < 30
### This number DOES NOT included unmapped endogenous reads
# echo "number of endogenous reads incorrectly mapped w mapq < 30"
# $9=="cat": reads are endogenous
# $4<30: mapping quality below 30
# $10==""FASLE": reads are mapped incorrectly (true_organism!=mapped_organism and/or true_record!
=mapped_rname and/or true_start!=mapped_pos)
# $5!="*": only mapped reads
Enb30F=$(awk '($9=="cat" && $4<30 && $10=="FALSE" && $5!="*"){print $0}' ${i}.TrueFalse.tab | wc -l)

### number of endogenous reads not mapped
### mapped_rname and mapped_organism = *
# echo "number of endogenous reads not mapped"
# $9=="cat": reads are endogenous
# $5=="*": read is unmapped ($5=mapped_organism)
# $"=="*": read is unmapped ($2=mapped_rname)
EnUn=$(awk '($9=="cat" && $2=="*" && $5=="*"){print $0}' ${i}.TrueFalse.tab | wc -l)

### exogenous reads
##############################################################
####

### number of exogenous reads mapped w min mapq 30
# echo "number of exogenous reads mapped w min mapq 30"
# $9!="cat" && $9!="true_organism": reads are exogenous
# $4>=30: mapping quality over 30
Exa30=$(awk '($9!="cat" && $9!="true_organism" && $4>=30){print $0}' ${i}.TrueFalse.tab | wc -l)

### number of exogenous reads correctly mapped w min mapq 30
### There are no correctly mapped exogenous reads; number should be zero
```

```
# echo "number of exogenous reads correctly mapped w min mapq 30"
# $9!="cat" && $9!="true_organism": reads are exogenous
# $4>=30: mapping quality over 30
# $10=="TRUE": reads are mapped correctly (true_organism=mapped_organism &&
true_record=mapped_rname && true_start=mapped_pos)
Exa30T=$(awk '($9!="cat" && $9!="true_organism" && $4>=30 && $10=="TRUE"){print $0}' $
{i}.TrueFalse.tab | wc -l)

### number of exogenous reads incorrectly mapped w min mapq 30
### These are all exogenous reads that have been mapped since exogenous reads cannot be correctly
mapped. (and unmapped reads do not have a mapq above 30)
# echo "number of exogenous reads incorrectly mapped w min mapq 30"
# $9!="cat" && $9!="true_organism": reads are exogenous
# $4>=30: mapping quality over 30
# $10==""FASLE": reads are mapped incorrectly (true_organism!=mapped_organism and/or true_record!
=mapped_rname and/or true_start!=mapped_pos)
# $5!="*": only mapped reads
Exa30F=$(awk '($9!="cat" && $9!="true_organism" && $4>=30 && $10=="FALSE" $$ $5!="*"){print $0}' $
{i}.TrueFalse.tab | wc -l)

### number of exogenous reads mapped w mapq < 30
### exogenous reads w mapq < 30 and unmapped exogenous reads
# echo "number of exogenous reads mapped w mapq < 30"
# $9!="cat" && $9!="true_organism": reads are exogenous
# $4<30: mapping quality below 30
Exb30=$(awk '($9!="cat" && $9!="true_organism" &&  $4<30){print $0}' ${i}.TrueFalse.tab | wc -l)

### number of exogenous reads correctly mapped w mapq < 30
### There are no correctly mapped exogenous reads; number should be zero
# echo "number of exogenous reads correctly mapped w mapq < 30"
# $9!="cat" && $9!="true_organism": reads are exogenous
# $4<30: mapping quality below 30
# $10=="TRUE": reads are mapped correctly (true_organism=mapped_organism &&
true_record=mapped_rname && true_start=mapped_pos)
Exb30T=$(awk '($9!="cat" && $9!="true_organism" && $4<30 && $10=="TRUE"){print $0}' $
{i}.TrueFalse.tab | wc -l)

### number of exogenous reads incorrectly mapped w mapq < 30
### These are incorrectly mapped exogenous reads DOES NOT include unmapped reads
#echo "number of exogenous reads incorrectly mapped w mapq < 30"
# $9!="cat" && $9!="true_organism": reads are exogenous
# $4<30: mapping quality below 30
# $10==""FASLE": reads are mapped incorrectly (true_organism!=mapped_organism and/or true_record!
=mapped_rname and/or true_start!=mapped_pos)
# $5!="*": only mapped reads
Exb30F=$(awk '($9!="cat" && $9!="true_organism" && $4<30 && $10=="FALSE" && $5!="*"){print $0}' $
{i}.TrueFalse.tab | wc -l)

### number of exogenous reads not mapped
### mapped_rname and mapped_organism = *
# echo "number of exogenous reads not mapped"
# $9!="cat" && $9!="true_organism": reads are exogenous
# $5=="*": read is unmapped ($5=mapped_organism)
# $"=="*": read is unmapped ($2=mapped_rname)
```

```
ExUn=$(awk '($9!="cat" && $9!="true_organism" && $2=="*" && $5=="*"){print $0}' ${i}.TrueFalse.tab | wc
-l)

echo "${i}	${En}	${Ena30}	${Ena30T}	${Ena30F}	${Enb30}	${Enb30T}
	${Enb30F}	${EnUn} ${Ex}	${Exa30}	${Exa30T}	${Exa30F}	${Exb30}
	${Exb30T}	${Exb30F}	${ExUn}"

done


head numbers_from_tab_w_HEADER.txt
runidx	En	Ena30	Ena30T	Ena30F	Enb30	Enb30T	Enb30F	EnUn	Ex	Exa30	Exa30T
	Exa30F	Exb30	Exb30T	Exb30F	ExUn
0	260000	210327	209910	417	49673	11425	11601	26647	740000	7245	0	7245
	732755	0	5564	727191
10	260000	184504	184055	449	75496	8074	10523	56899	740000	5694	0	5694
	734306	0	4257	730049
11	260000	181904	181523	381	78096	9783	10513	57800	740000	4415	0	4415
	735585	0	3119	732466
12	260000	178301	177939	362	81699	12222	10384	59093	740000	3114	0	3114
	736886	0	2600	734286
13	260000	167440	167164	276	92560	18437	10135	63988	740000	2183	0	2183
	737817	0	2046	735771
14	260000	154231	154023	208	105769	26261	9882	69626	740000	1784	0	1784
	738216	0	1906	736310
15	260000	114250	114141	109	145750	46826	9233	89691	740000	1111	0	1111
	738889	0	1579	737310
16	260000	92249	92174	75	167751	56152	8684	102915	740000	885	0	885
	739115	0	1498	737617
17	260000	81153	81084	69	178847	58162	8455	112230	740000	832	0	832
	739168	0	1442	737726
```

Code used to generate figures

Input: table “numbers_from_tab_w_HEADER.txt”, with added columns identifying the parameters mismatch values “n”, seed length “l” and runtime “CPU_time_total”.

```
### load libraries and packages
library(ggplot2)
library(devtools)
library(scales)

### disable scientific notation to avoid exponential scale labels
options(scipen = 999)

### Set working directory
setwd("path/to/input/")

### Read in table
*_numbers_tab<-read.table("*_numbers_from_tab_Header_n_l_time.csv", header=TRUE, sep=",", dec=".")

### Sensitivity ###
### Overlay unfiltered and filtered values (filtered for mapping quality with samtools view -q)
### Attention: Parsing after filtering does not reveal the correct total number of endogenous reads. The sensitivity after
filtering has to be related to the correct total number of endogenous reads!
```

```
### Attention: Numbers STILL CONTAIN DUPLICATES
### Numbers have been extracted from the *.TrueFalse.tab files ###
### Ena30T: endogenous reads mapped with mapq >= 30 (DOES NOT include unmapped exogenous reads since unmapped
reads cannot be correctly mapped)
### Enb30T: endogenous reads mapped with mapq < 30 (DOES NOT include unmapped exogenous reads since unmapped
reads cannot be correctly mapped)

plot(*_numbers_tab)+
  geom_bar(aes(x=factor(n), y=(Ena30T+Enb30T)/En, fill=factor(l), alpha=1/10), stat="identity", position = position_dodge())+
  geom_bar(aes(x=factor(n), y=Ena30T/En, fill=factor(l)), stat="identity", position = position_dodge(), colour="white")+
  labs(fill="seed length (l)")+
  ggtitle("Sensitivity depending on mismatch value (n) and seed length (l)")+
  xlab("mismatch value (n)")+
  ylab("sensitivity")+scale_y_continuous(limits=c(0,1), breaks=seq(0,1,1/10))+
  theme(axis.text.x=element_text(angle=90, vjust=0.5, size=12), axis.title=element_text(size=14),
axis.text.y=element_text(size=12), legend.justification =c(1,1), legend.position=c(1,1), legend.text=element_text(size=12),
legend.title = element_text(size=12))+guides(alpha=FALSE)


### False positives = mapped exogenous reads before and after filtering for mapping quality ###
### Overlay the ratio of (incorrectly) mapped exogenous reads before and after filtering (filtering for mapping quality with
samtools view -q)
### Attention: Numbers STILL CONTAIN DUPLICATES
### Numbers have been extracted from the *.TrueFalse.tab files ###
### False positives related to the total number of mapped reads
### En: total number of endogenous reads (input)
### Ex: total number of exogenous reads (input)
### EnUn: number of unmapped endogenous reads
### ExUn: number of unmapped exogenous reads
### Total number of mapped reads= En+Ex-EnUn-ExUn [UNFILTERED]
### Ena30: number of mapped endogenous reads with mapq >= 30 (both, correctly and incorrectly mapped reads)
### Exa30: number of mapped exogenous reads with mapq >= 30
### Total number of mapped reads= Ena30+Exa30 [FILTERED]

plot(*_numbers_tab)+
  geom_bar(aes(x=factor(n), y=(Exa30F+Exb30F)/(En+Ex-EnUn-ExUn), fill=factor(l), alpha=1/10), stat="identity", position =
position_dodge())+
  geom_bar(aes(x=factor(n), y=Exa30F/(Ena30+Exa30), fill=factor(l)), stat="identity", position = position_dodge(),
colour="white")+
  labs(fill="seed length (l)")+
  ggtitle("Ratio of mapped exogenous reads to the total number of mapped reads")+
  xlab("mismatch value (n)")+ylab("ratio mapped exogenous reads")+
  scale_y_continuous(limits=c(0,0.15), breaks=seq(0,1,1/50))+
  theme(axis.text.x=element_text(angle=90, size=12, vjust=0.5), axis.title=element_text(size=14),
axis.text.y=element_text(size=12), legend.justification =c(1,1), legend.position=c(1,1), legend.text=element_text(size=12),
legend.title=element_text(size=12))+guides(alpha=FALSE)

# Runtime ###
### bar chart n vs. runtime [s] bwa aln grouped by l
### Attention: This plot refers to the CPU time (not the realtime)
### Times are identical for unfiltered and filtered data
ggplot(*_numbers_tab)+
  geom_bar(aes(x=factor(n), y=CPU_time_total, fill=factor(l)), stat="identity", position=position_dodge())+
  xlab("mismatch value (n)")+
  ylab("CPU time [s]")+
  labs(fill="seed length (l)")+
  ggtitle("CPU runtime [s] depending on mismatch value (n) and seed length (l)")+
  theme(axis.text.x=element_text(angle=90, size=14), axis.title=element_text(size=16), axis.text.y=element_text(size=14),
legend.justification =c(1,1), legend.position=c(1,1), legend.text=element_text(size=14), legend.title=element_text(size =14))
```

References

1. Knapp, M.; Clarke, A. C.; Horsburgh, K. A.; Matisoo-Smith, E. A. Setting the stage – Building and working in an ancient DNA laboratory. *Ann. Anat. - Anat. Anz.* **2012**, *194*, 3–6, doi:10.1016/j.aanat.2011.03.008.
2. Rohland, N.; Siedel, H.; Hofreiter, M. Nondestructive DNA extraction method for mitochondrial DNA analyses of museum specimens. *BioTechniques* **2004**, *36*, 814–816, 818–821.
3. Dabney, J.; Knapp, M.; Glocke, I.; Gansauge, M.-T.; Weihmann, A.; Nickel, B.; Valdiosera, C.; García, N.; Pääbo, S.; Arsuaga, J.-L.; Meyer, M. Complete mitochondrial genome sequence of a Middle Pleistocene cave bear reconstructed from ultrashort DNA fragments. *Proc. Natl. Acad. Sci.* **2013**, *110*, 15758–15763, doi:10.1073/pnas.1314445110.
4. Gansauge, M.-T.; Meyer, M. Single-stranded DNA library preparation for the sequencing of ancient or damaged DNA. *Nat. Protoc.* **2013**, *8*, 737–748, doi:10.1038/nprot.2013.038.
5. Paijmans, J. L. A.; Baleka, S.; Henneberger, K.; Taron, U. H.; Trinks, A.; Westbury, M. V.; Barlow, A. Sequencing single-stranded libraries on the Illumina NextSeq 500 platform. *ArXiv171111004 Q-Bio* **2017**.
6. Martin, M. Cutadapt removes adapter sequences from high-throughput sequencing reads. *EMBnet.journal* **2011**, *17*, 10–12.
7. Li, H.; Durbin, R. Fast and accurate short read alignment with Burrows–Wheeler transform. *Bioinformatics* **2009**, *25*, 1754–1760, doi:10.1093/bioinformatics/btp324.
8. Li, H.; Handsaker, B.; Wysoker, A.; Fennell, T.; Ruan, J.; Homer, N.; Marth, G.; Abecasis, G.; Durbin, R. The Sequence Alignment/Map format and SAMtools. *Bioinformatics* **2009**, *25*, 2078–2079, doi:10.1093/bioinformatics/btp352.
9. Jónsson, H.; Ginolhac, A.; Schubert, M.; Johnson, P. L. F.; Orlando, L. mapDamage2.0: fast approximate Bayesian estimates of ancient DNA damage parameters. *Bioinformatics* **2013**, *29*, 1682–1684, doi:10.1093/bioinformatics/btt193.
10. Węcek, K.; Hartmann, S.; Paijmans, J. L. A.; Taron, U.; Xenikoudakis, G.; Cahill, J. A.; Heintzman, P. D.; Shapiro, B.; Baryshnikov, G.; Bunevich, A. N.; Crees, J. J.; Dobosz, R.; Manaserian, N.; Okarma, H.; Tokarska, M.; Turvey, S. T.; Wójcik, J. M.; Żyła, W.; Szymura, J. M.; Hofreiter, M.; Barlow, A. Complex Admixture Preceded and Followed the Extinction of Wisent in the Wild. *Mol. Biol. Evol.* **2017**, *34*, 598–612, doi:10.1093/molbev/msw254.
11. R Core Team *R: A Language and Environment for Statistical Computing*; R Foundation for Statistical Computing: Vienna, Austria, 2017;
