# Supplementary figures and images for: Testing of Alignment Parameters for Ancient Samples: Evaluating and Optimizing Mapping Parameters for Ancient Samples Using the TAPAS Tool
Source: Genes (Basel). 2018 Mar 13;9(3):157. doi: 10.3390/genes9030157 (PMC5867878; doi:10.3390/genes9030157)

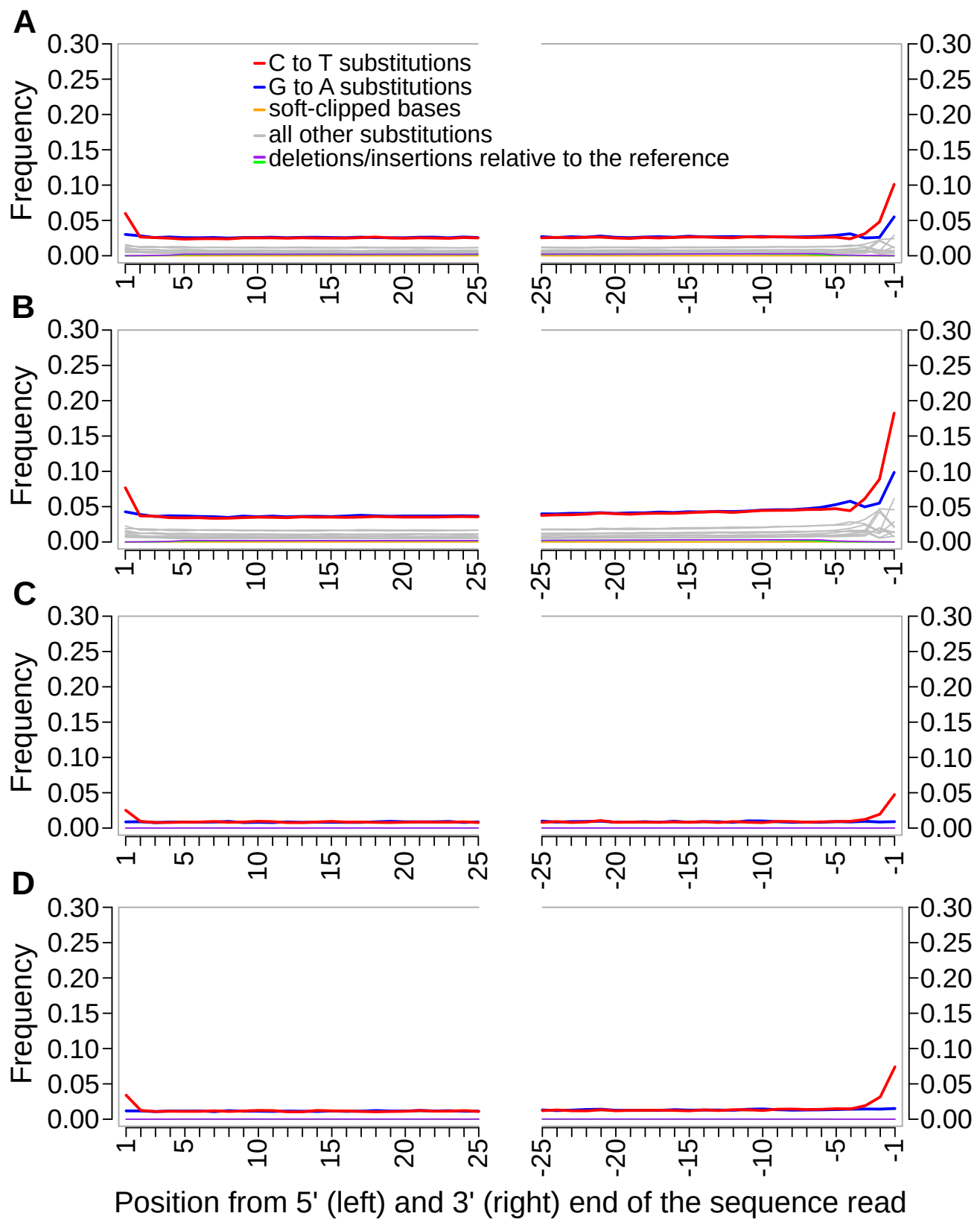

Supplement: Supplementary file 1 [file genes-09-00157-s001.zip › TAPAS_FigureS2_mapDamage_12Mar2018.pdf]

CPU runtime [s] depending mismatch value ( $n$ ) and seed length ( $l$ )

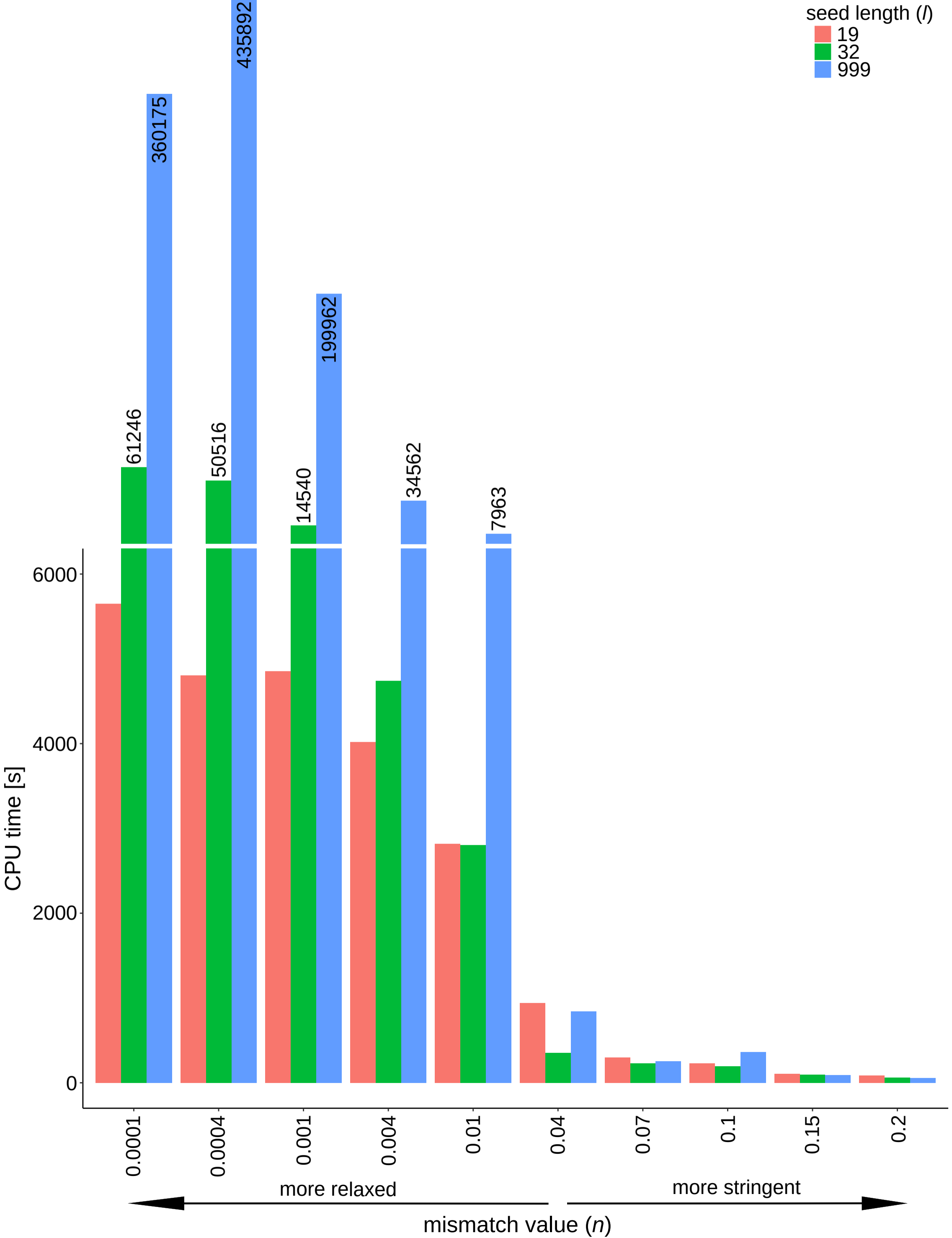

Supplement: Supplementary file 1 [file genes-09-00157-s001.zip › TAPAS_FigureS3_Runtime_Linsang_12Mar2018.pdf]
